# Prevalence and correlates of sexual violence against adolescents: Quantitative evidence from rural and urban communities in South-West Nigeria

**DOI:** 10.1371/journal.pgph.0004223

**Published:** 2025-02-11

**Authors:** Liliana Abreu, Tobias Hecker, Katharina Goessmann, Taiwo Oludare Abioye, Wasiu Olorunlambe, Anke Hoeffler

**Affiliations:** 1 Department of Politics and Public Administration, University of Konstanz, Konstanz, Germany; 2 Department of Psychology & Institute for Interdisciplinary Research on Conflict and Violence, Bielefeld University, Bielefeld, Germany; 3 Institute of Peace and Strategic Studies, University of Ibadan, Ibadan, Nigeria; St John’s Medical College, INDIA

## Abstract

Despite the recognized need to address the prevention of sexual violence against adolescents in Nigeria, significant research gaps persist in understanding the patterns, determinants, and impacts of such violence, particularly regarding regional variations and the specific developmental needs of adolescents across different stages. This study provides Nigerian regional prevalence estimates disaggregated by gender, rural/urban, and in/out-of-school populations, while also identifying socio-demographic and cultural determinants related to increased vulnerability. A cross-sectional survey was conducted in South-West Nigeria with a sample of 961 adolescents, targeting in- and out-of-school adolescents aged 13–17 years. Descriptive statistics and logistic regression analyses were performed.The prevalence of any form of SV since age 12 was 69.4%, with higher rates among out-of-school adolescents and boys. Non-contact abuse (63.2%), passive contact abuse (41.9%), and active contact abuse (28.7%) were the most common forms reported. Peers were the dominant perpetrators (77.1%), followed by other adults (27.9%). Being male (OR 2.033), older (OR 1.214 per year), involved in a romantic relationship (OR 2.731), and experiencing SV before age 12 (OR 4.622) were significant risk factors. Higher household wealth (OR 0.902 per asset) and emotional support from both parents (OR 0.413) were protective factors.This study highlights the high burden of SV against adolescents in Nigeria, with concerning patterns of male victimization and peer perpetration. The findings emphasize the need for comprehensive, evidence-based strategies addressing emotional support, social norms, power dynamics, and economic vulnerabilities to prevent and respond to this problem effectively.

## Introduction

Sexual violence (SV) against children and adolescents is a serious human rights violation and a critical public mental health concern with ramifications reverberating across the globe [1]. Adolescents, defined as individuals aged 10-19 years, are particularly vulnerable to sexual violence due to their developmental stage, power imbalances, and limited access to resources and support systems [2–4]. Additionally, other social cognitive and reward processes may increase adolescents sensitivity to social cues and peer influences [5–8], potentially increasing their vulnerability to sexual violence perpetrated by peers or in social contexts [4]. Consequently, the impact of SV during this critical period can have lifelong adverse effects, including a toll on mental, physical, and reproductive health, bearing potential consequences that drastically influence the individual’s trajectory [5–8]. Within adolescence, recent or ongoing SV has been associated with a range of psychological consequences, including sleep disturbances, suicidal ideation and persistent feelings of loneliness [9], depressive symptoms [10], post-traumatic stress disorders [11], as well as internalizing and externalizing behavior problems [12].

SV is particularly concerning in low- and middle-income countries (LMICs) like Nigeria, where children are more susceptible to experiencing adverse childhood experiences due to the high prevalence of poverty [13–17]. A meta-analysis conducted by Stoltenborgh and colleagues (2013) [18] indicated that Sub-Saharan African (SSA) countries may endure some of the highest rates of sexual violence against boys and girls during childhood and adolescence globally, with over 25% of adolescents affected [19,20]. In addition to the high estimates, the existing studies demonstrated a wide variation of prevalences (5%-38%) [21–24]. In Nigeria, the burden of sexual violence against adolescents is alarmingly high, yet often under-reported [25,26]. According to the Violence Against Children Survey (VACS) [21], six out of every ten children experienced some form of violence in Nigeria, with the highest rates reported in the South-South and North-Central regions [27]. Approximately half of Nigerian children reported experiencing some form of physical violence before the age of 18, whether from an intimate partner, parent, adult relative, or a member of their community [21]. In a systematic review, Beyene and colleagues observed rates of sexual coercion and abuse among adolescents Nigerian girls ranging between 11 to 55% [28]. Nevertheless, among the children who reported experiencing violence, fewer than five out of every 100 received any form of support [21] and fewer than 5% of girls who experience sexual violence in Nigeria actively seek help, with merely 14% being aware of where to seek assistance [21,29]. Ultimately, only an estimated 23% of reported rape cases reach trial [30]. In addition to the problem, these figures unlikely estimates the true magnitude of the problem, due to cultural stigma, fear of victim-blaming, and ineffective prosecution of perpetrators [31,32]. To address this problem, Nigeria made substantial commitments to addressing violence against children, and instituted the “Year of Action to End Violence Against Children” in 2016 [33]. However, complex mechanisms such as legal, cultural and social appear to indulge perpetration and under-reporting of SV among Nigerian adolescents [34].

Despite the gravity of the problem, significant research gaps persist in understanding the patterns, determinants, and impact of SV against adolescents in Nigeria. Most existing studies are hospital-based or localized, lacking comprehensive regional data and a wider understanding of regional variations [31,35]. Furthermore, there is a lack of research focused on male survivors of sexual violence, as well as on the unique developmental needs and long-term consequences for adolescent survivors [3].

Our study advances on prior evidence in two important ways. Specifically:

1. We provide regional estimates of the prevalence of sexual violence against adolescents, disaggregated by gender, age, rural and urban communities, and in-and-out of school adolescents.
2. We identify key socio-demographic, cultural, and environmental determinants that contribute to the vulnerability of adolescents to SV.

## Methods

### Study design and participants

The study included both direct contact violence and non-contact forms, while also examining the dynamics of the survivor-perpetrator relationship. We employed an adapted questionnaire [19] to conduct a comprehensive survey including 961 adolescents in the designated region. The survey was carried between 1^st^ May and 28^th^ August 2021.

Due to Nigeria’s size and ongoing political instability, nationally representative studies are challenging. Instead, we focused on the geo-political zone of the South-West, which includes the important economic centers of Lagos and Ibadan and is home to 22% of all Nigerians. Data provided by UNICEF indicates that approximately 92% of adolescents in the South West region of Nigeria complete their primary education, while approximately 85% proceed to complete their secondary education. This suggests that there is a significant proportion of adolescents in the region who are not completing their education, estimated to be between 8 and 15% [36]. A cross-sectional design was adopted in this study. Ekiti state was excluded due to the deteriorating public security situation. Both in- and out-of-school adolescents aged 13-17 were the target population.

*In-school adolescents* – a multistage stratified sampling method was adopted and data were collected in five South-West Nigerian States. 27 public secondary schools from rural and urban communities were randomly sampled: Oyo (= 5), Osun (= 6), Lagos (n = 5), Ogun (n = 6), and Ondo (n = 4). Based on the Ministry of Education’s list of registered public secondary schools in the South-West geo-political zone, we selected the sample by using generated numbers on each community (state, local government area, public secondary schools, and current level of study). In each school, a class teacher was assigned by the head teacher to assist in generating sample frames through the class registers from which we randomly selected the participants. Between 15 to 45 adolescents were interviewed (n = 862).

*Out-of-school adolescents* **–** Given the challenges of reaching adolescents who do not attend school, systematic sampling was not possible, thus we collected a convenience sample in street locations where these adolescents congregated. This included bus stations and markets where they sell small goods and carry loads for stallholders and customers, as well as where they sleep (abandoned buildings, under bridges) and at roadsides where charitable donations are provided. Due to this method, we reached a severely disadvantaged sample of adolescents (n = 99).

### Procedures

Following established international guidelines [37], independent Yoruba native speakers, experienced in research and translation, translated all measures from English to Yoruba and then back to English. SurveyToGo software was used for the data collection, using individual tablets (except with subsample of the out-of-school, see Strengths and Limitations for details).

#### Data collection.

Data were collected by four postgraduate students of social sciences at the University of Ibadan: two females and two males. The enumerators were trained on safety, ethical and scientific procedures, child protection, and confidentiality. An informed consent was given to all participants to be signed by their parents/legal guardians, where we assured that none of their socio-demographic information would be shared with third parties. To reduce social desirability bias, the questionnaire was self-administered with the enumerators present to clarify any questions if needed. The survey was carried out within the premises of the school and completion took an average of 35 minutes. For the out-of-school sample, informed consent was given, but we used a different strategy (see Ethical Considerations, for details). In accordance with legal and ethical standards for research involving children, we ensured compliance with relevant child protection legislation, obtained parental consent alongside adolescent consent, and maintained participant confidentiality throughout the study. Interviews with the out-of-school participants lasted on average longer than with in-school participants (50 minutes).

#### Main outcome variables.

The dependent variable, the experience of SV, was measured in five categories [19,38]:

1. Passive contact abuse, defined as being sexually touched by another person against one’s will.
2. Active contact abuse, defined as being forced to touch somebody else sexually.
3. Forced intercourse, which may include any type of penetrative sexual activity.
4. Non-contact abuse, defined as unwanted exposure to pornographic material, verbal harassment or dissemination of personal and intimate photos or messages on the internet.
5. Transactional abuse, defined as being coerced to perform sexual acts in exchange for money or goods, including forced prostitution.

If respondents confirmed the experience of a given sexual assault, they were also asked to indicate whether it was done to them once or several times and by whom (parent/adult living in the house; siblings/youth living in the house; teacher/school staff; peer at school; another adult; another peer). We also gathered information on whether the abuse happened only before the age of 12, only after the age of 12, or whether the SV occurred before and after the age of 12. In our analysis, we used the experiences of SV after the age of 12 as the dependent variable and the experience of SV before the age of 12 as an explanatory variable.

#### Independent variables.

We assessed correlations between SV and peer violence, which was measured using the Multidimensional Peer Victimization Scale (MPVS) [37]. MPVS is a self-report tool comprising 16 items designed to evaluate instances of physical and psychological violence experienced by peers within the last month, with demonstrated satisfactory psychometric properties [37] and previous validation for a Nigerian sample [38]. Other explanatory variables were socio-demographic characteristics of the participants such as gender, age, location (urban or rural area), living situation, and who provides emotional and financial support. Additionally, relationship status, levels of food and nutrition as well as economic welfare were assessed with a set of binary questions (yes/no) at the end of the questionnaire.

### Ethical considerations

We followed ethical guidelines as outlined by Mathews et al. (2022) [35]. The protocol was reviewed and approved by the Institute for Advanced Medical Research and Training (IAMRT), College of Medicine, University of Ibadan (UI/UCH number: UI/EC/21/0031) and the Institutional Review Board (Ethics Committee) of the University of Konstanz (IRB number: 21/2021). The study was conducted in accordance with the declaration of Helsinki. Data was collected with tablets and anonymized. Verbal and written consent were obtained from participants involved in this study. During data collection, following permission from head teachers, we explained the purpose of the study in the classroom and provided the adolescents with a parental consent form. On the following day, depending on individual and a signed parental consent, we carried out the survey. For out-of-school participants we could not obtain parental consent, because most do not live with their parents neither had a legal guardian. Almost all of these adolescents organize their lives independently, so it seemed appropriate to rely on their informed consent.

Enumerators received extensive training on scientific procedures of data collection, resilience, risk assessment and ethical protocols such as the need to ensure confidentiality, safety and privacy of the respondents. Moreover, they were trained and instructed to refer participating adolescents exhibiting marked psychological distress or who reported ongoing emotional, physical, or sexual abuse to the study psychologist who conducted a follow-up interview. A total of 53 adolescents were referred to either school counsellors or NGOs.

Enumerators as well as participants strictly adhered to the Covid-19 Protocols by wearing face masks. Refreshments (snacks) and 200 Naira (about $0.5) were given to each participant after the assessment, they had no prior knowledge of this small compensation of time and efforts.

Due to the sensitive nature of the topic we used gender-matching techniques, i.e., only female enumerators were responsible for guiding and interviewing the female participants and only male enumerators attended to the male participants.

#### Reflexivity statement.

Data collection was carried out by four recent postgraduate social science students from the University of Ibadan, who were specially trained for their role in this research. Their training focused on quantitative interviewing techniques, ethical considerations and child protection protocols. However, their training did not extend to manuscript writing or academic publication.

The involvement of local data enumerators was instrumental in sensitive cultural nuances and building rapport with participants. The lead local researcher (WO) adopted an informed approach led by his academic expertise (in psychology) and commitment to equity and ethical practice. Regular team discussions were held to reflect on how individual backgrounds, values and assumptions might shaped the research process, particularly in relation to the sensitive issues emerging during data collection. Finally, the team took steps to ensure the psychological well-being of data enumerators by providing self-care resources and ways for emotional support during and after fieldwork. These measures were aimed at preventing vicarious trauma and fostering a supportive research environment.

#### Legal considerations.

Adherence to legal and ethical standards in conducting research involving children was a priority for the research team. The study was designed and conducted in full compliance with the applicable legal frameworks, including the Child Rights Act and the Violence Against Persons Prohibition (VAPP) Act, which protect children from abuse, exploitation and other forms of violence. The research team ensured familiarity with these legal instruments and incorporated them into the study protocols. Specifically: (i) The research team and data collectors received training on the legal obligations associated with the study, including recognising and responding to potential cases of child sexual abuse or female genital mutilation (FGM). (ii) A clear protocol was established for reporting any identified cases of child sexual abuse, FGM or other forms of child rights violations to the appropriate legal and child protection authorities.

#### Statistical analysis.

The prevalence of SV experienced by the participating adolescents was calculated using descriptive analyses. Group differences for prevalence between gender and in- and out-of-school adolescents were determined with chi-squared tests. To determine possible contributing factors for SV, multiple logistic regression analyses were conducted producing odds ratios with 95%-confidence intervals and alpha levels of.05. SPSS 28 and Stata 15 were used for the analysis.

## Results

### Participants characteristics

In a total of 961 participants in Nigeria, we asked every participant how they identified regarding gender and all opted for binary gender identification (518 girls and 443 boys). The sample comprised adolescents aged 13 to 17, with 95.9% aged between 15 and 17 years. Table 1 provides a comprehensive overview of participants’ characteristics. Urban communities constituted a majority (75.7%) of the surveyed areas, and a distinct difference emerged in living arrangements between in-school and out-of-school adolescents. While 88.6% of in-school adolescents lived with either one or both parents, a significant majority (56.6%) of out-of-school adolescents did not reside with their parents. Financial support primarily came from parents for in-school adolescents (91.6%), with a notable contrast in out-of-school adolescents (48.5%). Emotional support was predominantly derived from one or both parents (88.6%), but this was markedly higher among in-school participants (92.8%) compared to out-of-school respondents (51.5%). Regarding romantic relationships, 57.6% of participating adolescents reported being in one. This percentage was substantially higher among out-of-school adolescents (76.8%) in comparison to their in-school counterparts (55.5%). A noteworthy 20.1% of adolescents reported an age gap of five or more years with their partners. A substantial proportion (43.8%) of adolescents indicated experiencing some form of SV before the age of 12, with a marked difference observed between in-school (41.4%) and out-of-school (64.6%) adolescents. Additionally, a wealth index was constructed based on household assets, showing that while the average adolescent lived in a household possessing 5.74 assets, the out-of-school group had significantly fewer assets (2.53 assets). Moreover, a substantial difference emerged in the proportion of households owning seven or more assets (9.1% for out-of-school versus 31.0% for in-school adolescents).

**Table 1:** Participants characteristics.

	Total Sample	In-school sample	Out-of-school sample	Significance Test
**Gender**	n %	n %	n %	
Female	518 53.9	482 55.9	36 36.4	T⁄²(1, n = 961) = 13.663; p < 0.001
Male	443 46.1	380 44.1	63 63.6	
**Age (in years; range 13–17)**	M=16.24, SD 0.89	M=16.27, SD 0.84	M=16.02, SD 1.25	t(108.339)= −1.927, p = 0.057.
**Age groups**				
Early adolescence (age 13 - 14)	39 4.1	8 3.2	11 11.1	
Middle adolescence (age 15 - 17)	922 95.9	834 96.8	88 88.9	
**Sexual Violence under 12 years**				
under 12 years	421 43.8	357 41.4	64 64.6	T⁄²(1, n = 961) = 19.468; p < 0.001
other	540 56.2	505 58.6	35 35.4	
**Setting**				
Urban	727 75.7	655 76.0	72 72.7	T⁄²(1, n = 961) = 0.512; p = 0.474
Rural	234 24.3	207 24.0	27 27.3	
**Living context**				
Live with 1 parent	288 30.0	259 30.0	29 29.3	T⁄²(2, n = 961) = 145.692; p < 0.001
Live with both parents	519 54.0	505 58.6	14 14.1	
Other	154 16.0	98 11.4	56 56.6	
**Financial support**				
parents	838 87.2	790 91.6	48 48.5	T⁄²(5, n = 961) = 324.880; p < 0.001
relatives	53 5.5	43 5.0	10 10.1	
any other caregiver or guardian	20 2.1	19 2.2	1 1.0	
government/organization (NGO)	7 0.7	6 0.7	1 1.0	
no one	41 4.3	4 0.5	37 37.4	
my boyfriend/girlfriend	2 0.2	0 0.0	n = 2 0.2	
**Are you in a relationship**				
With a peer	554 57.6	478 55.5	76 76.8	T⁄²(1, n = 961) = 16.525; p < 0.001
With a person >5 years older	193 20.1	170 19.7	23 23.2	T⁄²(1, n = 961) = 0.682; p = 0.409
**Household wealth**				
**Household assets(1 – 10)** ^**1**^	Mean = 5.40, SD = 1.986	Mean = 5.74, SD = 1.644	Mean = 2.53, SD = 2.358	t(109.216) = −13.185, p < 0.001.
**Emotional support**				
both parents	851 88.6	800 92.8	51 51.5	T⁄²(1, n = 961) = 149.376; p < 0.001
mother only				
father only				
Elsewhere	110 11.4	62 7.2	48 48.5	
TOTAL	961	862	99	

### Prevalence of sexual violence experiences

The prevalence of SV experiences among adolescents is presented in Table 2. The results indicated that a majority (69.4%) of adolescents experienced some form of SV since the age of 12. The spectrum of experiences ranged from non-contact abuse (63.2%) to passive contact abuse (41.9%), active contact abuse (28.7%), forced intercourse (21.3%), and transactional sex (14.9%). Substantial differences emerged between in-school and out-of-school adolescents, as well as between boys and girls. Out-of-school adolescents consistently reported higher instances of SV across all categories, while boys, irrespective of school attendance, reported significantly more SV experiences.

**Table 2:** Prevalences of sexual violence.

	Total	In-school	Out-of-school	In/Out school comparison	Male	Female	Gender comparison
	%	%	%	*p*	%	%	*p*
Any sexual violence^1^	69.4 (667)	68.0 (586)	81.8 (81)	8.007 (p < 0.01)**	81.5 (361)	59.1 (306)	56.509 (p < 0.001)***
Passive contact abuse^2^	41.9 (403)	39.7 (342)	61.6 (61)	17.557 (p < 0.001)***	54.6 (242)	31.1 (161)	54.371 (p < 0.001)***
Active contact abuse^3^	28.7 (276)	26.9 (232)	44.4 (44)	13.330 (p < 0.001)***	41.8 (185)	17.6 (91)	68.272 (p < 0.001)***
Forced intercourse^4^	21.3 (205)	18.8 (162)	43.4 (43)	32.129 (p < 0.001)***	27.3 (121)	16.2 (84)	17.524 (p < 0.001)***
Non-contact abuse^5^	63.2 (607)	61.3 (528)	79.8 (79)	13.126 (p < 0.001)***	77.4 (343)	51.0 (264)	71.860 (p < 0.001)***
Transactional^6^	14.9 (143)	13.3 (115)	28.3 (28)	15.652 (p < 0.001)***	17.2 (76)	12.9 (67)	3.360 (*p = 0.067)*^±^
Total:	961	862	99		443	518	

^1^ This refers to any form of sexual violence including contact, non-contact, transactional and forced intercourse as reported by adolescents aged 12 years and above.

^2^ Passive contact abuse means being unwillingly touched by someone in a sexual way as reported by adolescents aged 12 years and above.

^3^ Active contact abuse means being forced to touch someone in a sexual way as reported by adolescents aged 12 years and above.

^4^ Forced intercourse means any type of penetration (vaginal, anal, oral) as reported by adolescents aged 12 years and above.

^5^ Non-contact abuse means unwanted online exposure of own nude pictures, videos, or intimate messages on social media/internet) as reported by adolescents aged 12 years and above.

^6^ Transactional simply refers to payment in exchange for sex or any other related sexual activity as reported by adolescents aged 12 years and above.

*** p < 0.001, ** p < 0.01, * p < 0.05, ^±^ p < 0.1.

### Perpetration of sexual violence

When analysing perpetrators of sexual violence (SV) by gender, different patterns emerged. Peers were significant perpetrators for both males and females, but with notable differences in the specific types of peers involved.

#### Male survivors.

For male survivors, female peers emerged as the main perpetrators in most forms of SV (Table 3). Female peers at school (24.4%) and other female peers (23.2%) accounted for the highest percentages of perpetrators for all forms of sexual abuse. This pattern is particularly pronounced for active contact abuse, where female peers at school (37.3%) and other female peers (29.5%) are the dominant perpetrators. Male peers also play a significant role, especially in non-contact abuse (27.1% for male peers at school and 22.2% for other male peers). However, their involvement is generally lower than that of female peers. Adults, both male and female, are less frequently reported as perpetrators by male victims, with percentages typically ranging from 5% to 10% across different forms of abuse.

**Table 3:** Perpetrators of sexual violence disaggregated by gender (male and female).

Perpetrators	Any form of sexual abuse	Passive Contact Abuse	Active Contact Abuse	Forced Intercourse	Non-Contact Abuse	Transactional
	%	%	%	%	%	%
**Perpetrators of Sexual Violence disaggregated by gender (male)**						
Female parent/adult living in the home	6.0	8.8	5.2	7.6	4.2	10.0
Male parent/adult living in the home	5.2	6.4	5.2	5.2	3.7	12.7
Female sibling/child living in the home	4.5	3.6	5.2	5.8	3.7	8.2
Male sibling/child living in the home	4.5	2.8	4.1	3.5	5.1	6.4
Female teachers/ other school staff	1.3	2.4	0.5	2.3	0.6	2.7
Male teacher/ other school staff	0.6	0.0	1.0	1.2	0.7	0.0
Female peer at school	24.4	22.4	37.3	24.4	21.8	22.7
Male peer at school	20.5	21.6	11.4	7.6	27.1	11.8
Any other female adult	9.4	10.0	8.3	14.5	7.5	13.6
Any other male adult	5.7	3.6	2.1	4.7	7.5	6.4
Any other female peer	23.2	19.2	29.5	40.1	18.2	27.3
Any other male peer	15.5	13.2	8.8	4.1	22.2	7.3
Total	120.7%	114.0%	118.7%	120.9%	122.2%	129.1%
N	1433	250	193	172	708	110
**Perpetrators of Sexual Violence disaggregated by gender (female)**						
Female parent/adult living in the home	8.5	9.8	7.2	3.1	9.4	11.2
Male parent/adult living in the home	6.9	11.0	8.2	13.1	3.3	5.6
Female sibling/child living in the home	4.6	3.5	6.2	3.8	4.9	4.5
Male sibling/child living in the home	5.0	5.8	3.1	6.9	4.5	5.6
Female teachers/ other school staff	1.3	1.7	0.0	2.3	0.9	2.2
Male teacher/ other school staff	2.2	2.9	1.0	3.8	1.6	2.2
Female peer at school	12.1	7.5	15.5	5.4	15.1	13.5
Male peer at school	18.4	20.8	16.5	19.2	19.3	10.1
Any other female adult	6.5	5.8	6.2	6.2	6.8	6.7
Any other male adult	23.6	32.4	18.6	31.5	18.4	25.8
Any other female peer	13.5	9.8	20.6	3.1	14.6	22.5
Any other male peer	23.0	19.1	19.6	30.0	22.8	24.7
Total	125.6%	130.1%	122.7%	128.5%	121.6%	134.8%
N	914	173	97	130	425	89

#### Female survivors.

The pattern of perpetration for female survivors is very different (Table 3). Male adults and peers are reported as perpetrators more often than female perpetrators. All other male adults (23.6%) and all other male peers (23.0%) are the most commonly reported perpetrators of all forms of sexual abuse. This pattern is even more pronounced for forced sexual intercourse, where any other male adult (31.5%) and any other male peer (30.0%) are the main perpetrators. Male peers at school (18.4%) also feature prominently in various forms of abuse. Female peers play a less significant role in the perpetration of abuse against female victims than they do for male victims. However, female peers are still significant perpetrators of active contact abuse (15.5% for female peers at school and 20.6% for any other female peer).

#### Common patterns.

For both genders, the total percentages exceed 100%, indicating that multiple perpetrators are often involved in incidents of SV. This pattern of multiple perpetrators is consistent across all forms of abuse for both males and females. Teachers and school staff are less frequently reported as perpetrators for both genders, with percentages generally below 3%. However, there is a slight increase in male teachers and school staff as perpetrators for female victims in cases of forced intercourse (3.8%). Transactional abuse shows a more diverse range of perpetrators for both genders, with a higher involvement of adults living in the same home compared to other forms of abuse.

### Correlates of sexual violence

The associations between adolescents’ characteristics and SV experiences are explored in Table 4. The baseline model, including gender, age, location, intimate partnership, and SV before the age of 12, revealed significant associations. Being male, older, and involved in a romantic relationship were all linked to a higher SV risk. Remarkably, the strongest predictor of SV was experiencing SV before the age of 12 (OR 4.622), followed by being in a romantic relationship (OR 2.731). Boys faced a higher risk than girls (OR 2.033). Our analysis of additional potential correlates and their significance demonstrated that the baseline model accounted for the higher prevalence of SV among out-of-school adolescents (column 2). Household wealth (columns 3&4) emerged as a significant factor, with each additional household asset reducing the SV risk (OR 0.902). Notably, adolescents who reported receiving emotional support from both parents (column 5) experienced a lower risk of SV (OR 0.413).

**Table 4:** Correlates of sexual violence.

	All forms of abuse	All forms of abuse	All forms of abuse	All forms of abuse	All forms of abuse
Female/Male (0/1)	2.033***	2.009***	2.091***	2.174***	2.008***
	(1.461 – 2.828)	(1.443 – 2.797)	(1.500 – 2.914)	(1.554 – 3.041)	(1.441 – 2.799)
Age (years)	1.265**	1.283**	1.281**	1.272**	1.261**
	(1.066 – 1.501)	(1.079 – 1.526)	(1.078 – 1.521)	(1.071 – 1.510)	(1.062 –1.499)
Urban/Rural (0/1)	0.812	0.815	0.834	0.834	0.826
	(0.560 – 1.177)	(0.562 – 1.181)	(0.574 – 1.211)	(0.574 – 1.211)	(0.568 – 1.200)
Romantic relationship (0/1)	2.731***	2.677***	2.637***	2.711***	2.772***
	(1.995 – 3.738)	(1.951 – 3.673)	(1.923 – 3.615)	(1.977 – 3.717)	(2.021 – 3.802)
SV before 12 y.o. (0/1)	4.622***	4.557***	4.620***	4.737***	4.515***
	(3.235 – 6.603)	(3.187 – 6.516)	(3.231 – 6.606)	(3.306 – 6.787)	(3.155 – 6.460)
In-school (0/1)		0.742	–		
		(0.405 – 1.359)			
Wh wealth (sum score 0-7)			0.902*	–	
			(0.827 – 0.984)		
Wealth Index (0/1)				0.586**	–
				(0.415 – 0.828)	
Emotional Support Parent (0/1)					0.413** -

*Notes:* Odds ratios reported. Asterisks indicate statistical significance p < 0.05, ** p < 0.01, *** p < 0.001. Reference categories Female/Male (0/1): 1 = Male (reference), 0 = Female; Urban/Rural (0/1): 1 = Rural (reference), 0 = Urban; Romantic relationship (0/1): 0 = No relationship (reference), 1 = In a relationship; SV before 12 y.o. (0/1): 0 = No sexual violence before 12 (reference), 1 = Experienced sexual violence before 12. Sample size (N) = 961.

Regarding gender interactions in the regression model (S8 Table. *Urban/rural gender interactions - Appendix*), the urban/rural effect becomes significant and protective when the interaction is taken into account (OR 0.579). This suggests that, overall, living in a rural location is associated with a lower likelihood of experiencing sexual violence. The significant interaction (OR 2.529) indicates that this location effect differs by gender, with the protective effect of an urban location (for males) being reversed (for females) due to the interaction effect.

## Discussion

This study provides comprehensive insights into the prevalence, perpetrators, and correlates of sexual violence (SV) experienced by adolescents in Nigeria. The findings revealed a significant burden, with nearly 70% of adolescents reporting some form of SV since the age of 12. This prevalence was higher than previous estimates from Nigeria, which ranged from 11% to 55% [39,40]. It is crucial to note that these estimates may still be an under-representation of the actual burden, as SV is often under-reported due to cultural stigma, fear of victim-blaming, and ineffective prosecution of perpetrators [32,41,42].

The discrepancy of estimates highlights the need for more comprehensive, nationwide data to accurately capture the magnitude of the problem. The spectrum of SV experiences was diverse, ranging from non-contact abuse (63.2%) to forced intercourse (21.3%) and transactional sex (14.9%). These findings underscore the pervasive nature of SV in adolescents’ lives, including various forms of exploitation and abuse. Notably, out-of-school adolescents consistently reported higher rates of SV across all categories, aligning with previous research that links school dropout to increased vulnerability to sexual exploitation, namely by peers [43]. The high prevalence rates observed shows the persistent nature of SV in the lives of Nigerian adolescents and highlight the necessity for more detailed and inclusive measures in future research. Furthermore, the discrepancy of estimates can also be attributed to two other factors: First, our survey included comprehensive and sensitive questions considering a wide range of SV experiences (passive, active, contact, non-contact, transactional), not just SV as forced sexual intercourse. Second, our survey allowed participants to self-report their experiences. This approach might have reduced the likelihood of under-reporting due to shame or social desirability bias. Brener and colleagues conducted a literature review on self-reports of health-risk behaviors among adolescents and observed that self-reporting is influenced by both cognitive factors (e.g.,: comprehension; retrieval; decision-making; and response generation) and situational factors, such as the presence of others while responding to questions. Thus, increasing social desirability bias (that is, the tendency to under-report stigmatized behaviors), as well as perceptions of the level of privacy and confidentiality. They concluded that these factors do not affect the validity of self-reports [24].

### Perpetration

Our gender-disaggregated analysis revealed different patterns of perpetration for male and female survivors of sexual violence. While peers are important perpetrators for both genders, male survivors are more likely to report female peers as perpetrators, particularly in cases of active contact abuse. In contrast, female survivors predominantly reported male adults and peers as perpetrators, especially in cases of forced intercourse. These findings highlight the need for gender-specific approaches in sexual violence prevention and intervention strategies, with a particular focus on peer-to-peer dynamics and adult-youth interactions. Often neglected, peer victimization have proven acute and lifelong consequences on mental health [43], with an increased risk of depression [44], and anxiety disorders [45]. For our sample, Doerr et al.[2] showed that adolescents who experienced the highest levels of SV also exhibited strongest negative impact on their mental and physical health. The high rates of peer perpetration (77.1%) observed in our study have significant implications within the context of Nigerian social structures and adolescent development theories. This finding is consistent with the social learning theory, which theorises that adolescents may learn and internalise violent behaviours through observation and imitation of peers [46]. In the Nigerian context, where hierarchical social structures and gender inequalities persist, peer-perpetrated SV may reflect broader societal norms and power dynamics. Moreover, the social ecological model of adolescent development shows the pivotal role of peer influences during this formative period [47]. The high prevalence of peer perpetration indicates a need for interventions that target peer groups and social norms, as opposed to focusing exclusively on individual-level factors or adult perpetrators. Such an approach could include the implementation of peer education programmes, the provision of bystander intervention training, and the introduction of initiatives that promote healthy relationships and gender equality among adolescents [48].

### Gender disparity

A prominent observation from this study was the significant gender disparity in SV experiences, with boys consistently reporting higher rates of SV across all categories compared to girls. While the VACS study [21] presented prevalence rates of SV for boys, it is noteworthy that the academic literature has generally paid limited attention to boys’ experiences in Nigeria, as evidenced by Nguyen et al.’s study [29]. The higher reported rates of SV among boys in our study prompt us to consider the need for a more detailed understanding of this issue, which has predominantly focused on female survivors. Many factors might influence this discrepancy, such as, cultural norms and reporting biases. In many Nigerian contexts, traditional gender roles may discourage boys from reporting SV due to fear of stigma or being perceived as weak, which could potentially lead to under-reporting in other studies [49,50]. Additionally, it is possible that societal norms, which emphasise male dominance and aggression, may contribute to the normalisation of SV against boys, which in turn may make it less likely to be recognised or reported as an abuse [51]. This emphasises the pressing need for gender-specific interventions that address these cultural barriers and promote a supportive environment for all survivors, regardless of gender [50]. Thus, it would be beneficial for policies to place an emphasis on increasing awareness about male victimisation and providing targeted support services that are sensitive to the unique challenges faced by male survivors.

### Correlates of risk and protective factors

The correlates of SV experiences showed key risk and protective factors. Being male, older, and involved in a romantic relationship were all associated with a higher risk of SV, aligning with previous research on vulnerability factors [31]. Remarkably, the strongest predictor was experiencing SV before the age of 12, suggesting a cyclical pattern of victimization and the need for an early intervention and support services.

On the other side, household wealth emerged as a significant factor, with higher socioeconomic status acting as a protective factor against SV. This finding underscores the role of poverty and economic vulnerability in exacerbating the risk of transactional abuse, particularly in resource-limited communities [31,52]. Previous studies disagreed on the importance of wealth. Yahaya et al. [53] found no correlation between socioeconomic status and childhood sexual abuse, while Miller and colleagues [54] suggested that children aged 13-17 years in households with high economic status are more likely to experience SV. Our data provided a more detailed insight. We found that although children from poorer households were more likely to experience SV, there was a threshold effect. The risk of SV is only significantly lowered for those from relatively wealthy households (with seven or more household assets, which applies to 28.7% of the adolescents interviewed).

Additionally, emotional support from both parents was associated with a lower risk of SV, highlighting the importance of family support systems and positive parenting practices in mitigating adolescents’ vulnerability. Therefore, in line with previous research [55], our results emphasize the impact of both parents emotional support correlated with lower probabilities of experiencing SV, transcending the significance of wealth in preventing SV. It may be beneficial to consider programmes that enhance parental involvement and communication, with the aim of fostering a supportive home environment where adolescents feel safe to discuss their experiences and seek help [56]. Furthermore, encouraging positive parenting practices and providing resources for parents to recognise signs of SV, could further enhance these protective effects [57].

This study also revealed that out-of-school adolescents consistently reported higher frequencies of SV across all categories compared to their in-school counterparts, and reported the highest rates of (poly)victimization. This included an elevated risk for non-contact abuse, contact abuse, and forced intercourse when compared to both in-school girls and boys. Few studies presented similar findings [58,59]. One study involving South African students revealed a higher prevalence of sexual violence among boys, with rates of 60% for boys compared to 53.7% for girls [59]. Targeted outreach and non-formal education programmes can be developed to reach out-of-school adolescents, especially in underserved communities. These programmes could offer flexible learning options, vocational training and life skills education tailored to their needs and circumstances. For example, the Association for Reproductive and Family Health (ARFH) in Nigeria has conducted outreach activities targeting out-of-school youth, including apprentices and artisans, providing training to empower them and help them make informed decisions about their sexual health [60]. In addition, accelerated learning programmes, such as those supported by Education Cannot Wait in northeast Nigeria, can help out-of-school adolescents catch up on missed education and potentially reintegrate into formal schooling [61]. Community-based interventions such as the WARIF Educational School Programme (WESP) could be adapted to reach out-of-school youth, focusing on building capacity to respond appropriately to and prevent sexual and gender-based violence [62]. In addition, integrating sexual and reproductive health education into these programmes, as demonstrated by projects such as the Life Planning Education Project, can address critical health needs of this vulnerable population [60]. These initiatives are a collaboration between government agencies, NGOs and community organisations, targeting the development of comprehensive strategies that address the multiple challenges faced by out-of-school adolescents, including poverty, early marriage and lack of access to health services.

### Urban-rural dynamics and gender interactions

Our analysis revealed complex interactions between gender and urban-rural setting in relation to sexual violence risk. When taking gender interactions into account, living in an urban area emerged as a protective factor against sexual violence (OR 0.579). However, this protective effect varied significantly by gender (interaction OR 2.529). The results suggest that the urban environment may be more protective for boys, while the protective effect may be reduced or possibly reversed for girls. This nuanced relationship highlights the importance of considering gender-specific experiences in different geographical contexts. Urban areas may offer more protection to boys through better access to education, support services and potentially more progressive social norms. Conversely, girls may face unique risks in urban settings, such as increased exposure to diverse social interactions, potential anonymity in larger populations, or specific forms of gender-based violence that are more prevalent in urban settings. These findings are consistent with previous research that has highlighted the complex interplay between urbanisation, gender and violence [63,64].

It is important to keep in mind that any correlates of intra- and interpersonal factors with violence victimisation/perpetration and derived patterns of violence need to be seen in context. In order to better understand the relevance of such factors for individual children and adolescents and their environment, we need more contextualising research approaches such as qualitative in-depth interviews and focus groups discussions.

### Patterns of violence

The analysis of different forms of SV revealed diverse patterns. For instance, boys faced higher risks of passive contact, active contact, and non-contact SV. The data indicated that older adolescents reported a greater number of experiences of both active and non-contact abuse. It is important to note, however, that this observation may be attributed to the cumulative nature of our measures (older, longer the exposure) rather than indicating increased vulnerability with age. The presence of a romantic partner amplified the risk across all SV forms, underscoring the potential for intimate partner violence. There was a substantial prevalence of non-contact abuse across the sampled groups: in-school girls (54%), in-school boys (72%), out-of-school girls (80%), and out-of-school boys (92%). This observation aligns with previous research by Rostad et al. [65] and Peter and Valkenburg [66], indicating a prominent link between pornography exposure and SV perpetration and victimization. This is particularly concerning in the digital age, where adolescents are increasingly exposed to online risks.

### Strengths and limitations

The study’s strengths include its regional representativeness, inclusion of both in- and out-of-school adolescents, and the use of validated measures and rigorous data collection procedures. In addition, various forms of SV were discerned through more detailed categorization: non-contact SV and contact SV, further disaggregated into passive and active contact abuse, forced intercourse, and transactional sex. However, it is important to acknowledge some limitations. First, the cross-sectional nature of the study excludes the establishment of causal relationships between the identified risk factors and SV experiences. Second, the out-of-school subsample was relatively small, and it was collected in very challenging circumstances. Due to safety concerns for the enumerators, data from the out-of-school adolescents was collected using pencil and paper rather than tablets, therefore these questionnaires were not self-reported. Asking face-to-face may have led to different disclosure rates, although existing evidence on this matter is inconclusive [67,68]. Third, requesting parental consent could potentially have led to under-reporting of SV situations carried out by parents or other family members. Finally, another limitation of our study is the lack of formal power calculations to determine sample size, which we strongly recommend for future research. Instead, our sample size was based on practical considerations, resource constraints and logistical factors, while aiming to reflect the population distribution of in-school and out-of-school youth in the study area. However, the present findings can guide future studies in their power calculations.

## Conclusion

This study revealed a significant burden of SV experienced by adolescents in Nigeria. Boys reported higher rates of victimization than girls, challenging the common perception that sexual violence primarily affects girls. The identified correlates, including gender, age, intimate partnerships, prior victimization, household wealth, and parental support, provide valuable insights for tailoring interventions to address the unique vulnerabilities and protective factors.

Future research should focus on longitudinal studies to better understand the long-term consequences of SV on adolescents’ physical, mental, and social well-being. Additionally, qualitative research can provide valuable insights into the lived experiences of survivors, their coping mechanisms, and the sociocultural factors that perpetuate or mitigate SV. In terms of practice, there is a pressing need to enhance the availability and accessibility of survivor-centered support services, including medical care, mental health support, and legal assistance. Establishing robust referral systems and ensuring the confidentiality and safety of survivors are priority. Additionally, capacity-building initiatives for healthcare professionals, law enforcement personnel, and other service providers are necessary to ensure a trauma-informed and sensitive approach to addressing the needs of SV survivors, regardless of gender.

## Supporting information

S1 Table Goodness of fit tests. (DOCX)

S2 Table Measure 2: Hosmer and Lemeshow test. (DOCX)

S3 Table Measure 3: Classification table. (DOCX)

S4 Table Correlates of sexual abuse. (DOCX)

S5 Table Correlates of sexual violence by type of abuse. (DOCX)

S6 Table Different forms of sexual violence. (DOCX)

S7 Table Perpetrators of sexual violence (full sample). (DOCX)

S8 Table Urban/rural gender interactions. (DOCX)

S1 Data Complete dataset. (7Z)

S2 Data Table reports. (XLSX)

S1 Checklist Inclusivity in global research. (DOCX)
