# Supplementary material for: Prevalence and correlates of sexual violence against adolescents: Quantitative evidence from rural and urban communities in South-West Nigeria
Source: PLOS Glob Public Health. 2025 Feb 11;5(2):e0004223. doi: 10.1371/journal.pgph.0004223 (PMC11813094; doi:10.1371/journal.pgph.0004223)
Supplement: S1 Table — (DOCX) [file pgph.0004223.s001.docx]

# S1 Table Goodness of fit tests (Baseline model, Table S1)

**Measure 1: Omnibus Tests of Model Coefficients**

| **Omnibus Tests of Model Coefficients** | | | | |
| --- | --- | --- | --- | --- |
|  |  |  |  |  |
|  | | Chi-square | df | Sig. |
| Step 1 | Step | 213.667 | 7 | <.001 |
|  | Block | 213.667 | 7 | <.001 |
|  | Model | 213.667 | 7 | <.001 |

The chi-square test result indicates that our model is a significant improvement in fit relative to an intercept-only model, χ² (7) = 213.667, p<.001.
