# Supplementary material for: Prevalence and correlates of sexual violence against adolescents: Quantitative evidence from rural and urban communities in South-West Nigeria
Source: PLOS Glob Public Health. 2025 Feb 11;5(2):e0004223. doi: 10.1371/journal.pgph.0004223 (PMC11813094; doi:10.1371/journal.pgph.0004223)
Supplement: S2 Table — (DOCX) [file pgph.0004223.s002.docx]

**S2 Table. Measure 2: Hosmer and Lemeshow Test (not significant test indicates we have evidence of an adequately specified model)**

| **Hosmer and Lemeshow Test** | | | |
| --- | --- | --- | --- |
| Step | Chi-square | Df | Sig. |
| 1 | 10.527 | 8 | .230 |

The Hosmer & Lemeshow test provides a second global fit test, testing the ‘estimated model to one that has perfect fit’ (Pituch & Stevens, 2015, p. 455). If this test is not significant, then we have evidence of an adequately specified model. If it is significant, then we have evidence that the model is misspecified, such as through omission of ‘nonlinear and/or interaction terms’ (p. 456). Here, we see the Hosmer and Lemeshow test is not statistically significant [χ² (8) = 10.527, p=.230], indicating an adequate fit of the model.
