# Supplementary material for: Prevalence and correlates of sexual violence against adolescents: Quantitative evidence from rural and urban communities in South-West Nigeria
Source: PLOS Glob Public Health. 2025 Feb 11;5(2):e0004223. doi: 10.1371/journal.pgph.0004223 (PMC11813094; doi:10.1371/journal.pgph.0004223)
Supplement: S3 Table — (DOCX) [file pgph.0004223.s003.docx]

**S3 Table. Measure 3: Classification Table (Higher sensitivity and specificity indicate a better fit of the model)**

| **Classification Table^a^** | | | | |
| --- | --- | --- | --- | --- |
| Observed | | Predicted | | |
|  |  | Sexual Violence over 12 | | Percentage Correct |
|  |  | 0 | 1 |  |
| Sexual Violence over 12 | 0 | 140 | 154 | 47.6 |
|  | 1 | 74 | 593 | 88.9 |
| Overall Percentage | |  |  | 76.3 |

The overall percentage indicates the percentage of cases with an observed outcome that were correctly predicted (in terms of the outcome) by the model. In this output, the overall percentage is 76.3%, computed as 100 %[( 140+593) / (140+154+74+593)] = 100% [733 / 961] = 76.3%.

Sensitivity refers to the percentage of cases observed to fall in the target group (Y=1; e.g., observed as having passed the test) correctly predicted by the model to fall into that group (e.g., predicted SV).

The sensitivity for the model is calculated as 100% [593/ (74+593)] = 100% [593/667] = 88.9%.

Specificity refers to percentage of cases observed to fall into the non-target (or reference) category who were correctly predicted by the model to fall into that group (e.g., predicted not abused).

The specificity for this model is calculated as 100% [140/ (140+154)] = 100% [112/244] = 47.6%.

Overall, the accuracy rate was 76.3%. The model exhibits good sensitivity since among those abused, 88.9% were predicted correctly to be abused based on the model. However, the model is weaker on predicting no abuse, only 47.6% of the non-abuse cases were correctly predicted.
