# Supplementary material for: Prevalence and correlates of sexual violence against adolescents: Quantitative evidence from rural and urban communities in South-West Nigeria
Source: PLOS Glob Public Health. 2025 Feb 11;5(2):e0004223. doi: 10.1371/journal.pgph.0004223 (PMC11813094; doi:10.1371/journal.pgph.0004223)
Supplement: S4 Table — (DOCX) [file pgph.0004223.s004.docx]

# S4 Table. Correlates of Sexual Abuse

|  | **1** | **2** | **3** | **4** | **5** | **6** | **7** | **8** |
| --- | --- | --- | --- | --- | --- | --- | --- | --- |
| Female/Male (0/1) | 2.033*** | 2.009*** | 2.091*** | 2.174*** | 2.012*** | 2.003*** | 2.008*** | 2.076*** |
|  | (1.461 - 2.828) | (1.443 - 2.797) | (1.500 - 2.914) | (1.554 - 3.041) | (1.445 - 2.802) | (1.438 - 2.789) | (1.441 - 2.799) | (1.490 - 2.892) |
| Age (years) | 1.265** | 1.283** | 1.281** | 1.272** | 1.255** | 1.257** | 1.261** | 1.275** |
|  | (1.066 - 1.501) | (1.079 - 1.526) | (1.078 - 1.521) | (1.071 - 1.510) | (1.057 - 1.490) | (1.059 - 1.492) | (1.062 - 1.499) | (1.074 - 1.513) |
| Urban/Rural (0/1) | 0.812 | 0.815 | 0.834 | 0.834 | 0.812 | 0.816 | 0.826 | 0.803 |
|  | (0.560 - 1.177) | (0.562 - 1.181) | (0.574 - 1.211) | (0.574 - 1.211) | (0.559 - 1.178) | (0.562 - 1.184) | (0.568 - 1.200) | (0.553 - 1.166) |
| In sexual relationship (0/1) | 2.731*** | 2.677*** | 2.637*** | 2.711*** | 2.747*** | 2.710*** | 2.772*** | 2.650*** |
|  | (1.995 - 3.738) | (1.951 - 3.673) | (1.923 - 3.615) | (1.977 - 3.717) | (2.006 - 3.763) | (1.979 - 3.711) | (2.021 - 3.802) | (1.933 - 3.634) |
| Prior sexual violence (0/1) | 4.622*** | 4.557*** | 4.620*** | 4.737*** | 4.559*** | 4.526*** | 4.515*** | 4.537*** |
|  | (3.235 - 6.603) | (3.187 - 6.516) | (3.231 - 6.606) | (3.306 - 6.787) | (3.189 - 6.518) | (3.165 - 6.472) | (3.155 - 6.460) | (3.171 - 6.489) |
| inschool (0/1) |  | 0.742 |  |  |  |  |  |  |
|  |  | (0.405 - 1.359) |  |  |  |  |  |  |
| hh wealth (sum score) |  |  | 0.902* |  |  |  |  |  |
|  |  |  | (0.827 - 0.984) |  |  |  |  |  |
| Wealth Index (0/1) |  |  |  | 0.586** |  |  |  |  |
|  |  |  |  | (0.415 - 0.828) |  |  |  |  |
| Living with Parent (0/1) |  |  |  |  | 0.66 |  |  |  |
|  |  |  |  |  | (0.415 - 1.051) |  |  |  |
| Financial Support Parents (0/1) |  |  |  |  |  | 0.628 |  |  |
|  |  |  |  |  |  | (0.365 - 1.082) |  |  |
| Emotional Support Parent (0/1) |  |  |  |  |  |  | 0.413** |  |
|  |  |  |  |  |  |  | (0.226 - 0.756) |  |
| Emotional Support 1 Parent (0/1) |  |  |  |  |  |  |  | 1.448* |
|  |  |  |  |  |  |  |  | (1.050 - 1.997) |

Notes: *** p<0.001, ** p<0.01, * p<0.05, ± p < 0.1
