# Supplementary material for: Prevalence and correlates of sexual violence against adolescents: Quantitative evidence from rural and urban communities in South-West Nigeria
Source: PLOS Glob Public Health. 2025 Feb 11;5(2):e0004223. doi: 10.1371/journal.pgph.0004223 (PMC11813094; doi:10.1371/journal.pgph.0004223)
Supplement: S5 Table — (DOCX) [file pgph.0004223.s005.docx]

# S5 Table. Correlates of Sexual Violence by Type of Abuse

| **Panel A** | **Passive contact abuse²** | **Active contact abuse³** | **Foced intercourse⁴** | **Non-contact abuse⁵** | **Transactional⁶** |
| --- | --- | --- | --- | --- | --- |
| female/male (0/1) | 0.646*** | 0.862*** | 0.133 | 0.821*** | -0.121 |
|  | 0.151 | 0.164 | 0.18 | 0.16 | 0.203 |
| age (years) | 0.004 | 0.096 | 0.185 | 0.163* | -0.044 |
|  | 0.086 | 0.097 | 0.108 | 0.085 | 0.113 |
| rural/ urban (0/1) | 0.127 | 0.221 | 0.31 | -0.164 | 0.337 |
|  | 0.171 | 0.178 | 0.188 | 0.182 | 0.207 |
| in sexual relationship (0/1) | 1.124*** | 0.951*** | 1.101*** | 0.818*** | 1.044*** |
|  | 0.156 | 0.177 | 0.205 | 0.155 | 0.235 |
| prior sexual violence (0/1) | 1.149*** | 1.070*** | 1.300*** | 1.570*** | 1.173*** |
|  | 0.148 | 0.162 | 0.184 | 0.167 | 0.209 |
|  |  |  |  |  |  |
| **Panel B** | **Passive contact abuse²** | **Active contact abuse³** | **Foced intercourse⁴** | **Non-contact abuse⁵** | **Transactional⁶** |
| female/male (0/1) | 0.621*** | 0.840*** | 0.051 | 0.801*** | -0.187 |
|  | 0.152 | 0.165 | 0.184 | 0.16 | 0.206 |
| age (years) | 0.026 | 0.112 | 0.237* | 0.187* | -0.007 |
|  | 0.087 | 0.097 | 0.109 | 0.087 | 0.114 |
| rural/ urban (0/1) | 0.126 | 0.217 | 0.304 | -0.159 | 0.333 |
|  | 0.171 | 0.178 | 0.191 | 0.183 | 0.209 |
| in sexual relationship (0/1) | 1.093*** | 0.927*** | 1.036*** | 0.786*** | 0.995*** |
|  | 0.157 | 0.178 | 0.207 | 0.156 | 0.236 |
| prior sexual violence (0/1) | 1.127*** | 1.051*** | 1.258*** | 1.560*** | 1.140*** |
|  | 0.149 | 0.163 | 0.186 | 0.168 | 0.211 |
| in school (0/1) | -0.442 | -0.316 | -0.911*** | -0.5 | -0.606* |
|  | 0.245 | 0.244 | 0.245 | 0.296 | 0.262 |
|  |  |  |  |  |  |
| Notes: |  |  |  |  |  |
| ¹ This refers to any form of sexual violence including contact, non-contact, transactional and forced intercourse as reported by adolescent aged 12 years and above | | | | | |
| ² Passive contact abuse means to being unwillingly touched by someone in a sexual way as reported by adolescent aged 12 years and above | | | | | |
| ³ Active contact abuse means being forced to touch someone in a sexual way as reported by adolescent aged 12 years and above . | | | | | |
| ⁴ Forced intercourse means any type of penetration (vaginal, anal, oral) as reported by adolescent aged 12 years and above | | | | |  |
| ⁵ Non-contact abuse means unwanted online exposure of own nude pictures, videos, or intimate messages on social media / internet) as reported by adolescent aged 12 years and above . | | | | | |
| ⁶ Transactional simply refers to payment in exchange for sex or any other related sexual activity as reported by adolescent aged 12 years and above . | | | | | |
| *** p<0.001, ** p<0.01, * p<0.05, ± p < 0.1 | | |  |  |  |
