# Supplementary material for: Prevalence and correlates of sexual violence against adolescents: Quantitative evidence from rural and urban communities in South-West Nigeria
Source: PLOS Glob Public Health. 2025 Feb 11;5(2):e0004223. doi: 10.1371/journal.pgph.0004223 (PMC11813094; doi:10.1371/journal.pgph.0004223)
Supplement: S6 Table — (DOCX) [file pgph.0004223.s006.docx]

S6 Table. **Different Forms of Sexual Violence**

|  | Passive contact abuse² | Active contact abuse³ | Foced intercourse⁴ | Non-contact abuse⁵ | Transactional⁶ | |
| --- | --- | --- | --- | --- | --- | --- |
| female/male (0/1) | 1.862*** | 2.315*** | 1.053 | 2.229*** | 0.829 |  |
|  | (1.383 - 2.506) | (1.675 - 3.200) | (0.735 - 1.508) | (1.629 - 3.049) | (0.554 - 1.243) |  |
| age (years) | 1.027 | 1.119 | 1.268* | 1.206* | 0.993 |  |
|  | (0.866 - 1.218) | (0.925 - 1.353) | (1.024 - 1.569) | (1.017 - 1.429) | (0.795 - 1.240) |  |
| urban/rural (0/1) | 1.134 | 1.243 | 1.356 | 0.853 | 1.395 |  |
|  | (0.811 - 1.587) | (0.876 - 1.763) | (0.933 - 1.970) | (0.596 - 1.221) | (0.927 - 2.100) |  |
| in sexual relationship (0/1) | 2.985*** | 2.526*** | 2.818*** | 2.149*** | 2.704*** |  |
|  | (2.195 - 4.058) | (1.784 - 3.578) | (1.879 - 4.225) | (1.617 - 2.978) | (1.703 - 4.295) |  |
| prior sexual violence (0/1) | 3.088*** | 2.859*** | 3.517*** | 4.713*** | 3.128*** |  |
|  | (2.306 - 4.134) | (2.079 - 3.933) | (2.444 - 5.060) | (3.394 - 6.545) | (2.070 - 4.726) |  |
| in school (0/1) | 0.643 | 0.729 | 0.402*** | 0.607 | 0.545* |  |
|  | (0.398 - 1.039) | (0.452 - 1.177) | (0.249 - 0.650) | (0.340 - 1.083) | (0.326 - 0.911) |  |

**Different Forms of Sexual Violence:** Further exploration of correlates across different forms of SV (e.g. *passive contact SV,* *active contact SV*, *forced intercourse, non-contact, and transactional abuse – see definitions in section 3.2.3*) is presented in Supplementary Materials. While out-of-school adolescents experienced higher SV rates, educational status was only significant in the analysis of forced intercourse. For passive, active, and non-contact SV, boys faced higher risks than girls (gender dummy: ORs 1.862, 2.315, and 2.229, respectively). Age was significant in active contact abuse and non-contact abuse instances, indicating a higher risk among older adolescents. As in the analysis of SV presented in Table 3, location was not significant. The presence of a romanticpartner amplified the risk across all SV forms (ORs 1.617 to 2.195). This was also the case for prior SV experiences (ORs range from 2.070 to 3.394
