# Supplementary material for: Prevalence and correlates of sexual violence against adolescents: Quantitative evidence from rural and urban communities in South-West Nigeria
Source: PLOS Glob Public Health. 2025 Feb 11;5(2):e0004223. doi: 10.1371/journal.pgph.0004223 (PMC11813094; doi:10.1371/journal.pgph.0004223)
Supplement: S7 Table — (DOCX) [file pgph.0004223.s007.docx]

S7 Table. Perpetrators of Sexual Violence (Full Sample)

| Perpetrators | Any form of sexual abuse | Passive Contact Abuse | Active Contact Abuse | Forced Intercourse | Non-Contact Abuse | Transactional |
| --- | --- | --- | --- | --- | --- | --- |
|  | % | % | % | % | % | % |
| Female parent/adult living in the home | 7.0 | 9.2 | 5.9 | 5.6 | 6.2 | 10.6 |
| Male parent/adult living in the home | 5.9 | 8.3 | 6.2 | 8.6 | 3.5 | 9.5 |
| Female sibling/child living in the home | 4.5 | 3.5 | 5.5 | 5.0 | 4.1 | 6.5 |
| Male sibling/child living in the home | 4.7 | 4.0 | 3.8 | 5.0 | 4.9 | 6.0 |
| Female teachers/ other school staff | 1.3 | 2.1 | 0.3 | 2.3 | 0.7 | 2.5 |
| Male teacher/ other school staff | 1.2 | 1.2 | 1.0 | 2.3 | 1.1 | 1.0 |
| Female peer at school | 19.6 | 16.3 | 30.0 | 16.2 | 19.2 | 18.6 |
| Male peer at school | 19.7 | 21.3 | 13.1 | 12.6 | 24.2 | 11.1 |
| Any other female adult | 8.2 | 8.3 | 7.6 | 10.9 | 7.2 | 10.6 |
| Any other male adult | 12.7 | 15.4 | 7.6 | 16.2 | 11.6 | 15.1 |
| Any other female peer | 19.4 | 15.4 | 26.6 | 24.2 | 16.9 | 25.1 |
| Any other male peer | 18.4 | 15.6 | 12.4 | 15.2 | 22.4 | 15.1 |
| Total | 122.6% | 120.6% | 120.0% | 124.1% | 122.0% | 131.7% |
| N | 2347 | 423 | 290 | 302 | 1133 | 199 |
