# Supplementary material for: Prevalence and correlates of sexual violence against adolescents: Quantitative evidence from rural and urban communities in South-West Nigeria
Source: PLOS Glob Public Health. 2025 Feb 11;5(2):e0004223. doi: 10.1371/journal.pgph.0004223 (PMC11813094; doi:10.1371/journal.pgph.0004223)
Supplement: S8 Table — (DOCX) [file pgph.0004223.s008.docx]

S8 Table: Urban/rural gender interactions

|  | Female only (n=518) | Male only (n=443) | Full sample |
| --- | --- | --- | --- |
| Female/Male (0/1) |  |  | 1.653** |
|  |  |  | (1.141 - 2.393) |
| Age (years) | 1.167 | 1.317** | 1.228** |
|  | (0.857 - 1.587) | (1.072 - 1.619) | (1.069 - 1.503) |
| Urban/Rural (0/1) | 1.474 | 0.574* | 0.579* |
|  | (0.770 - 2.821) | (0.357 - 0.922) | (0.361 - 0.927) |
| Romantic relationship (0/1) | 2.744*** | 2.718*** | 2.711*** |
|  | (1.598 - 4.714) | (1.841 - 4.013) | (1.978 - 3.715) |
| SV before 12 y.o. (0/1) | 4.514*** | 4.595*** | 4.552*** |
|  | (2.583 - 7.886) | (2.879 - 7.336) | (3. 183 - 6.508) |
| Interaction Gender*Location |  |  | 2.529* |
|  |  |  | (1.135 - 5.633) |
| *Notes:*  Odds ratios reported. Asterisks indicate statistical significance p < 0.05, ** p < 0.01, *** p < 0.001. Reference categories: Female/Male (0/1): 1 = Male (reference), 0 = Female; Urban/Rural (0/1): 1 = Rural (reference), 0 = Urban; Romantic relationship (0/1): 0 = No relationship (reference), 1 = In a relationship; SV before 12 y.o. (0/1): 0 = No sexual violence before 12 (reference), 1 = Experienced sexual violence before 12. Sample size (N) = 961. | | | |
